# Poly[[hexa­kis­(μ-benzene-1,4-dicarboxyl­ato)octa­kis­(*N*,*N*-dimethyl­acetamide)­hexa­manganese(II)] monohydrate]

**DOI:** 10.1107/S1600536811020010

**Published:** 2011-06-04

**Authors:** Ying Zhang, Chao-Xia Chu, Yi-Zhi Li

**Affiliations:** a School of Material Science and Engineering, Jiangsu University of Science and Technology, Zhenjiang 212003, People’s Republic of China; b Coordination Chemistry Institute and the State Key Laboratory of Coordination Chemistry, Nanjing University, Nanjing 210093, People’s Republic of China

## Abstract

In the title compound, {[Mn_6_(C_8_H_4_O_4_)_6_(C_4_H_9_NO)_8_]·H_2_O}_*n*_, two of the Mn atoms are six-coordinated by six O atoms from three benzene-1,4-dicarboxyl­ate (bdc) ligands and two *trans* DMA (dimethyl­acetamide) mol­ecules, whereas two other Mn atoms, located on inversion centers, are both in octa­hedral coordinations by six bdc O atoms. The discrete trinuclear manganese secondary building units (SBU) of Mn_3_(O_2_C*R*)_6_ ({–Mn—Mn—Mn-}) are linked through bdc ligands, forming a chain, while the discrete trinuclear SBU of {–Mn—Mn—Mn-} are bridged, forming another chain]. The two types of chains are linked through bdc ligands, resulting in the formation of a layer with 3^6^ topology. Weak O—H⋯O and O—H⋯N hydrogen-bonding inter­actions involving the disordered water molecule (half-occupation) extend the two-dimensional layers into a three-dimensional supra­molecular framework.

## Related literature

For related structures, see: Hawxwell *et al.* (2006 ▶); He *et al.* (2006 ▶); Williams *et al.* (2005 ▶). For general background to porous materials, see: Li *et al.* (2009 ▶).
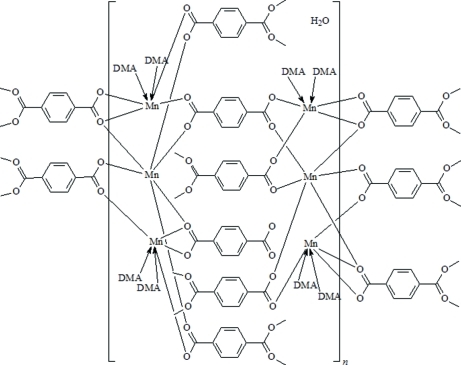


## Experimental

### 

#### Crystal data

[Mn_6_(C_8_H_4_O_4_)_6_(C_4_H_9_NO)_8_]·H_2_O
*M* *_r_* = 2029.30
Triclinic, 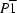

*a* = 9.924 (9) Å
*b* = 14.533 (13) Å
*c* = 16.990 (16) Å
α = 69.947 (13)°
β = 86.549 (14)°
γ = 83.807 (14)°
*V* = 2288 (4) Å^3^
*Z* = 1
Mo *K*α radiation
μ = 0.89 mm^−1^
*T* = 291 K
0.18 × 0.16 × 0.14 mm

#### Data collection

Bruker SMART APEX CCD diffractometer
Absorption correction: multi-scan (*SADABS*; Bruker, 2004 ▶) *T*_min_ = 0.776, *T*_max_ = 0.815
18098 measured reflections
8890 independent reflections
6477 reflections with *I* > 2σ(*I*)
*R*_int_ = 0.044

#### Refinement

*R*[*F* ^2^ > 2σ(*F* ^2^)] = 0.058
*wR*(*F* ^2^) = 0.121
*S* = 1.04
8890 reflections
591 parameters
H-atom parameters constrained
Δρ_max_ = 0.30 e Å^−3^
Δρ_min_ = −0.42 e Å^−3^

### 

Data collection: *SMART* (Bruker, 2004 ▶); cell refinement: *SAINT* (Bruker, 2004 ▶); data reduction: *SAINT*; program(s) used to solve structure: *SHELXTL* (Sheldrick, 2008 ▶); program(s) used to refine structure: *SHELXTL*; molecular graphics: *DIAMOND* (Brandenburg, 2006 ▶); software used to prepare material for publication: *SHELXTL*.

## Supplementary Material

Crystal structure: contains datablock(s) I, global. DOI: 10.1107/S1600536811020010/bx2348sup1.cif

Structure factors: contains datablock(s) I. DOI: 10.1107/S1600536811020010/bx2348Isup2.hkl

Additional supplementary materials:  crystallographic information; 3D view; checkCIF report

## Figures and Tables

**Table 1:** Hydrogen-bond geometry (Å, °)

*D*—H⋯*A*	*D*—H	H⋯*A*	*D*⋯*A*	*D*—H⋯*A*
O17—H17*B*⋯O16^i^	0.85	2.61	3.236 (7)	131
O17—H17*B*⋯N4^i^	0.85	2.62	3.463 (8)	170

Symmetry code: (i) 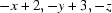.

**Fig. 1.**
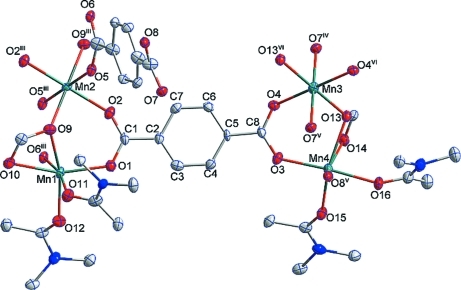
ORTEP diagram of the title compound. Displacement ellipsoids are drawn at 30% probability level. Hydrogen atoms, solvent water molecules are omitted for clarity. Symmetry codes: (iii) -x + 1, -y + 1, -z + 1; (iv) x + 1, y, z; (v) -x + 1, -y + 2, -z; (vi) 2 - x, 2 - y, -z.

**Fig. 2.**
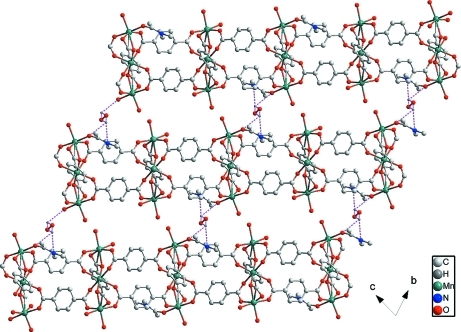
The three-dimensional supramolecular network of the title compound.

## supplementary crystallographic information

### Comment

Metal-organic frameworks (MOFs) are currently under massive investigation due to their fascinating properties and high potential as a new class of porous materials (Li *et al.*, 2009). Our particular interest is to construct three-dimensional (three-dimensional) frameworks with more open porosity using benzene-1,4-dicarboxylicacid (H_2_bdc) and pillar bidentate ligands as mixed ligands, and to probe the influence of pillar ligands on the topological networks. Recently, we have employed the pillar bidentate ligand 4,4'-azopyridine. Unexpectedly, a new MOF without the pillared ligand, {[Mn_6_(bdc)_6_(DMA)_8_].H_2_O}*_n_*, has been obtained.

The title compound contains two types of crystallographically equivalent six-coordinated Mn centers, and bdc ligands adopt two coordination modes. Mn1 and Mn4 atoms are both six-coordinated to six oxygen atoms from three bdc ligands and two *trans*-DMA molecules,whereas Mn2 and Mn3 located on an inversion center are both in octahedral coordination with six oxygen atoms of bdc species. The average bond distances of Mn1—O, Mn2—O, Mn3—O, and Mn4—O are 2.213, 2.167, 2.146, and 2.207 Å, respectively. The discrete trinuclear manganese secondary building unit (SBU) of Mn_3_(O_2_CR)_6_ ({–Mn4—Mn3—Mn4-}) are linked through bdc ligands to form a one-dimensional chain, while the discrete trinuclear SBU of {–Mn1—Mn2—Mn1-} are bridged to form another one-dimensional chain.Two types of one-dimensional chains are linked through bdc ligands, resulting in the formation of a two-dimensional layer with 3^6^ topology. The 3^6^ net also displays an interdigitated structure, with the interplanar distance between adjacent nets being 7.043 Å. The structure of the 3^6^ layer has been also observed in other MOFs (Hawxwell *et al.*, 2006; He *et al.*, 2006; Williams *et al.*, 2005).The weak hydrogen-bonding interactions extend the two-dimensional layers into a three-dimensional supramolecular framework.

### Experimental

A mixture of MnCl_2_.4H_2_O (0.0198 g, 0.1 mmol), benzene-1,4-dicarboxylic acid (0.0166 g, 0.1 mmol), 4,4'-azopyridine (0.0184 g, 0.1 mmol) combined with 6 ml DMA was stirred for 20 min at room temperature. Then the solution was heated solvothermally in a 25 ml Teflon-lined stainless-steel vessel at 160 *^o^*C for 72 h under autogenous pressure. Slow cooling of the resulting solution to room temperature at the rate of 10 *^o^*C.h^-1^ afforded colorless block-shaped crystals suitable for single-crystal X-ray structure analysis. Yield based on based on MnCl_2_.4H_2_O: 41%.

### Refinement

All non-hydrogen atoms were refined with anisotropic thermal parameters. The H atoms of H_2_bdc ligands and DMA molecules were calculated at idealized positions with C—H = 0.93 or 0.96 Å and included in the refinement in a riding mode with *U*_iso_ for H assigned as 1.2 or 1.5 times *U*_eq_ of the attached atoms. The water molecule is disordered and the H atoms bound to oxygen atoms from water molecules were located from difference maps and refined as riding, with O - H restraint (O - H = 0.85 Å), and with *U*_ĩso_(H) = 1.2*U*_eq_(O).

### Crystal data

**Table:**

[Mn_6_(C_8_H_4_O_4_)_6_(C_4_H_9_NO)_8_]·H_2_O	*Z* = 1
*M**_r_* = 2029.30	*F*(000) = 1048
Triclinic, *P*1	*D*_x_ = 1.473 Mg m^−^^3^
Hall symbol: -P 1	Mo *K*α radiation, λ = 0.71073 Å
*a* = 9.924 (9) Å	Cell parameters from 1310 reflections
*b* = 14.533 (13) Å	θ = 2.4–26.1°
*c* = 16.990 (16) Å	µ = 0.89 mm^−^^1^
α = 69.947 (13)°	*T* = 291 K
β = 86.549 (14)°	Block, colorless
γ = 83.807 (14)°	0.18 × 0.16 × 0.14 mm
*V* = 2288 (4) Å^3^	

### Data collection

**Table:**

Bruker SMART APEX CCD diffractometer	8890 independent reflections
Radiation source: sealed tube	6477 reflections with *I* > 2σ(*I*)
graphite	*R*_int_ = 0.044
φ and ω scans	θ_max_ = 26.0°, θ_min_ = 1.5°
Absorption correction: multi-scan (*SADABS*; Bruker, 2004)	*h* = −12→12
*T*_min_ = 0.776, *T*_max_ = 0.815	*k* = −17→17
18098 measured reflections	*l* = −20→20

### Refinement

**Table:**

Refinement on *F*^2^	Primary atom site location: structure-invariant direct methods
Least-squares matrix: full	Secondary atom site location: difference Fourier map
*R*[*F*^2^ > 2σ(*F*^2^)] = 0.058	Hydrogen site location: inferred from neighbouring sites
*wR*(*F*^2^) = 0.121	H-atom parameters constrained
*S* = 1.04	*w* = 1/[σ^2^(*F*_o_^2^) + (0.05*P*)^2^ + 1.55*P*] where *P* = (*F*_o_^2^ + 2*F*_c_^2^)/3
8890 reflections	(Δ/σ)_max_ < 0.001
591 parameters	Δρ_max_ = 0.30 e Å^−^^3^
0 restraints	Δρ_min_ = −0.42 e Å^−^^3^

### Special details

**Table:**

Geometry. All e.s.d.'s (except the e.s.d. in the dihedral angle between two l.s. planes) are estimated using the full covariance matrix. The cell e.s.d.'s are taken into account individually in the estimation of e.s.d.'s in distances, angles and torsion angles; correlations between e.s.d.'s in cell parameters are only used when they are defined by crystal symmetry. An approximate (isotropic) treatment of cell e.s.d.'s is used for estimating e.s.d.'s involving l.s. planes.
Refinement. Refinement of *F*^2^ against ALL reflections. The weighted *R*-factor *wR* and goodness of fit *S* are based on *F*^2^, conventional *R*-factors *R* are based on *F*, with *F* set to zero for negative *F*^2^. The threshold expression of *F*^2^ > σ(*F*^2^) is used only for calculating *R*-factors(gt) *etc*. and is not relevant to the choice of reflections for refinement. *R*-factors based on *F*^2^ are statistically about twice as large as those based on *F*, and *R*- factors based on ALL data will be even larger.

### Fractional atomic coordinates and isotropic or equivalent isotropic displacement parameters (Å^2^)

**Table:**

	*x*	*y*	*z*	*U*_iso_*/*U*_eq_	Occ. (<1)
C1	0.6079 (4)	0.7147 (3)	0.4638 (2)	0.0346 (8)	
C2	0.6860 (4)	0.7813 (3)	0.3964 (2)	0.0346 (8)	
C3	0.6914 (4)	0.8782 (3)	0.3875 (2)	0.0345 (8)	
H3	0.6454	0.9032	0.4262	0.041*	
C4	0.7653 (4)	0.9408 (3)	0.3207 (2)	0.0371 (9)	
H4	0.7677	1.0060	0.3171	0.045*	
C5	0.8349 (4)	0.9085 (3)	0.2602 (2)	0.0351 (9)	
C6	0.8252 (4)	0.8097 (3)	0.2667 (2)	0.0337 (8)	
H6	0.8635	0.7869	0.2247	0.040*	
C7	0.7595 (4)	0.7463 (3)	0.3350 (2)	0.0363 (8)	
H7	0.7629	0.6800	0.3411	0.044*	
C8	0.9124 (4)	0.9703 (3)	0.1889 (2)	0.0364 (9)	
C9	0.4520 (4)	0.5199 (3)	0.3057 (2)	0.0386 (9)	
C10	0.3845 (4)	0.5940 (3)	0.2290 (2)	0.0371 (9)	
C11	0.3482 (4)	0.6902 (3)	0.2222 (2)	0.0351 (8)	
H11	0.3649	0.7119	0.2659	0.042*	
C12	0.2865 (4)	0.7553 (3)	0.1505 (2)	0.0397 (9)	
H12	0.2686	0.8214	0.1448	0.048*	
C13	0.2517 (4)	0.7233 (3)	0.0880 (2)	0.0419 (10)	
C14	0.2938 (4)	0.6252 (3)	0.0947 (2)	0.0366 (8)	
H14	0.2759	0.6038	0.0511	0.044*	
C15	0.3606 (4)	0.5598 (3)	0.1634 (2)	0.0344 (8)	
H15	0.3887	0.4955	0.1663	0.041*	
C16	0.1706 (4)	0.7945 (3)	0.0147 (2)	0.0416 (9)	
C17	0.2092 (3)	0.5711 (3)	0.5685 (2)	0.0293 (7)	
C18	0.1036 (4)	0.5320 (3)	0.5330 (2)	0.0343 (8)	
C19	−0.0124 (4)	0.5022 (3)	0.5807 (3)	0.0354 (8)	
H19	−0.0208	0.5025	0.6355	0.042*	
C20	−0.1171 (3)	0.4715 (3)	0.5462 (2)	0.0298 (7)	
H20	−0.1960	0.4535	0.5776	0.036*	
C21	0.3282 (4)	0.9478 (3)	0.4694 (2)	0.0412 (9)	
H21A	0.3563	0.9315	0.5260	0.062*	
H21B	0.2683	1.0072	0.4544	0.062*	
H21C	0.4064	0.9572	0.4324	0.062*	
C22	0.2578 (4)	0.8677 (3)	0.4624 (3)	0.0379 (9)	
C23	0.2436 (4)	0.9462 (3)	0.3088 (3)	0.0481 (10)	
H23A	0.2964	0.9927	0.3184	0.072*	
H23B	0.1601	0.9794	0.2832	0.072*	
H23C	0.2937	0.9163	0.2722	0.072*	
C24	0.1468 (4)	0.7890 (3)	0.3833 (3)	0.0435 (10)	
H24A	0.2042	0.7291	0.4061	0.065*	
H24B	0.1284	0.7988	0.3260	0.065*	
H24C	0.0631	0.7849	0.4152	0.065*	
C25	0.2922 (4)	0.6604 (3)	0.8043 (3)	0.0445 (10)	
H25A	0.2166	0.6584	0.7724	0.067*	
H25B	0.2596	0.6734	0.8542	0.067*	
H25C	0.3461	0.5982	0.8194	0.067*	
C26	0.3779 (4)	0.7410 (3)	0.7521 (2)	0.0315 (8)	
C27	0.5451 (4)	0.8643 (3)	0.7399 (3)	0.0434 (9)	
H27A	0.5713	0.8470	0.6911	0.065*	
H27B	0.6229	0.8552	0.7736	0.065*	
H27C	0.5086	0.9319	0.7231	0.065*	
C28	0.3830 (4)	0.8082 (3)	0.8706 (2)	0.0412 (9)	
H28B	0.2909	0.8371	0.8631	0.062*	
H28C	0.4358	0.8486	0.8886	0.062*	
H28A	0.3853	0.7437	0.9123	0.062*	
C29	1.2530 (4)	1.0724 (3)	0.0613 (2)	0.0365 (8)	
C30	1.3790 (4)	1.0338 (3)	0.0268 (2)	0.0371 (9)	
C31	1.3923 (4)	1.0379 (3)	−0.0530 (3)	0.0465 (10)	
H31	1.3212	1.0660	−0.0894	0.056*	
C32	1.5108 (4)	1.0008 (3)	−0.0814 (3)	0.0385 (9)	
H32	1.5184	0.9985	−0.1355	0.046*	
C33	0.9785 (4)	1.1317 (3)	0.3481 (3)	0.0408 (9)	
H33A	0.8963	1.1589	0.3678	0.061*	
H33B	1.0349	1.0959	0.3948	0.061*	
H33C	0.9571	1.0881	0.3201	0.061*	
C34	1.0555 (4)	1.2154 (3)	0.2870 (2)	0.0329 (8)	
C35	1.1307 (4)	1.2506 (3)	0.4156 (3)	0.0444 (10)	
H35A	1.0619	1.2960	0.4278	0.067*	
H35B	1.2174	1.2616	0.4312	0.067*	
H35C	1.1111	1.1845	0.4468	0.067*	
C36	1.2147 (5)	1.3460 (3)	0.2672 (3)	0.0519 (11)	
H36B	1.2841	1.3569	0.2992	0.078*	
H36C	1.1564	1.4056	0.2449	0.078*	
H36A	1.2557	1.3266	0.2220	0.078*	
C37	1.3693 (5)	1.3544 (4)	0.0014 (3)	0.0568 (12)	
H37B	1.4274	1.2952	0.0078	0.085*	
H37C	1.3615	1.3665	0.0537	0.085*	
H37A	1.4072	1.4087	−0.0408	0.085*	
C38	1.2266 (5)	1.3429 (3)	−0.0251 (3)	0.0486 (11)	
C39	1.0383 (4)	1.3542 (3)	−0.1284 (3)	0.0498 (11)	
H39A	1.0317	1.2859	−0.1195	0.075*	
H39B	1.0189	1.3924	−0.1857	0.075*	
H39C	0.9742	1.3754	−0.0921	0.075*	
C40	1.2821 (5)	1.4090 (4)	−0.1795 (3)	0.0574 (12)	
H40A	1.3513	1.4363	−0.1597	0.086*	
H40B	1.2364	1.4594	−0.2249	0.086*	
H40C	1.3227	1.3571	−0.1986	0.086*	
Mn1	0.38729 (5)	0.67699 (4)	0.60045 (3)	0.02763 (13)	
Mn2	0.5000	0.5000	0.5000	0.02486 (16)	
Mn3	1.0000	1.0000	0.0000	0.03000 (18)	
Mn4	1.04197 (6)	1.17403 (4)	0.11125 (4)	0.03636 (15)	
N1	0.2148 (3)	0.8716 (2)	0.3871 (2)	0.0361 (7)	
N2	0.4408 (3)	0.8006 (2)	0.7893 (2)	0.0409 (8)	
N3	1.1331 (4)	1.2662 (3)	0.3229 (2)	0.0433 (8)	
N4	1.1816 (4)	1.3683 (3)	−0.1093 (2)	0.0491 (9)	
O1	0.5369 (3)	0.75104 (19)	0.51206 (16)	0.0370 (6)	
O2	0.6154 (3)	0.62445 (19)	0.47181 (16)	0.0381 (6)	
O3	0.9342 (3)	1.05421 (19)	0.19333 (16)	0.0362 (6)	
O4	0.9591 (2)	0.93877 (18)	0.13152 (15)	0.0334 (6)	
O5	0.4543 (3)	0.55091 (18)	0.36912 (15)	0.0343 (6)	
O6	0.4982 (2)	0.43460 (17)	0.30840 (14)	0.0297 (5)	
O7	0.1522 (3)	0.88310 (18)	0.01153 (17)	0.0379 (6)	
O8	0.1204 (3)	0.75779 (19)	−0.03211 (16)	0.0386 (6)	
O9	0.3222 (2)	0.59198 (18)	0.52522 (15)	0.0328 (6)	
O10	0.1904 (3)	0.58840 (19)	0.63579 (16)	0.0366 (6)	
O11	0.2320 (3)	0.79224 (19)	0.52664 (17)	0.0398 (6)	
O12	0.3879 (3)	0.7629 (2)	0.68427 (17)	0.0417 (6)	
O13	1.1432 (3)	1.09305 (19)	0.02173 (15)	0.0358 (6)	
O14	1.2535 (3)	1.0920 (2)	0.13129 (18)	0.0428 (7)	
O15	1.0527 (3)	1.2349 (2)	0.21012 (17)	0.0399 (6)	
O16	1.1533 (3)	1.2991 (2)	0.03719 (19)	0.0491 (7)	
O17	0.9587 (6)	1.4716 (4)	0.0090 (4)	0.0522 (15)	0.50
H17C	0.9436	1.4145	0.0420	0.063*	0.50
H17B	0.9335	1.5153	0.0310	0.063*	0.50

### Atomic displacement parameters (Å^2^)

**Table:**

	*U*^11^	*U*^22^	*U*^33^	*U*^12^	*U*^13^	*U*^23^
C1	0.0317 (19)	0.044 (2)	0.0288 (19)	−0.0172 (16)	0.0065 (15)	−0.0105 (17)
C2	0.038 (2)	0.034 (2)	0.0314 (19)	−0.0140 (16)	0.0023 (15)	−0.0073 (15)
C3	0.0338 (19)	0.039 (2)	0.0322 (19)	0.0067 (16)	−0.0075 (15)	−0.0159 (16)
C4	0.039 (2)	0.0303 (19)	0.044 (2)	−0.0090 (16)	−0.0003 (17)	−0.0137 (17)
C5	0.038 (2)	0.036 (2)	0.0310 (19)	−0.0189 (16)	0.0197 (16)	−0.0092 (16)
C6	0.0366 (19)	0.039 (2)	0.0289 (19)	−0.0033 (16)	0.0031 (15)	−0.0158 (16)
C7	0.038 (2)	0.0321 (19)	0.043 (2)	−0.0094 (16)	−0.0059 (17)	−0.0151 (17)
C8	0.038 (2)	0.046 (2)	0.0260 (19)	−0.0159 (17)	0.0108 (15)	−0.0112 (17)
C9	0.0332 (19)	0.041 (2)	0.034 (2)	0.0148 (17)	−0.0179 (16)	−0.0063 (17)
C10	0.0317 (19)	0.042 (2)	0.033 (2)	0.0166 (16)	−0.0166 (16)	−0.0103 (17)
C11	0.047 (2)	0.039 (2)	0.0239 (18)	−0.0092 (17)	−0.0022 (16)	−0.0143 (16)
C12	0.053 (2)	0.0242 (18)	0.042 (2)	−0.0118 (17)	0.0065 (18)	−0.0091 (16)
C13	0.040 (2)	0.047 (2)	0.030 (2)	0.0169 (18)	−0.0143 (16)	−0.0050 (17)
C14	0.041 (2)	0.038 (2)	0.036 (2)	−0.0060 (17)	−0.0061 (16)	−0.0170 (17)
C15	0.047 (2)	0.0268 (18)	0.0255 (18)	−0.0022 (16)	0.0051 (16)	−0.0047 (14)
C16	0.045 (2)	0.043 (2)	0.035 (2)	0.0160 (18)	−0.0237 (18)	−0.0132 (18)
C17	0.0257 (17)	0.0306 (18)	0.0253 (17)	0.0081 (14)	−0.0146 (14)	−0.0024 (14)
C18	0.0374 (19)	0.0318 (19)	0.035 (2)	−0.0164 (16)	−0.0102 (16)	−0.0073 (15)
C19	0.0318 (19)	0.037 (2)	0.040 (2)	−0.0054 (16)	−0.0108 (16)	−0.0154 (17)
C20	0.0265 (17)	0.0313 (18)	0.0290 (18)	0.0036 (14)	−0.0068 (14)	−0.0077 (15)
C21	0.042 (2)	0.044 (2)	0.036 (2)	−0.0166 (18)	−0.0012 (17)	−0.0071 (18)
C22	0.0312 (19)	0.032 (2)	0.048 (2)	0.0113 (15)	−0.0188 (17)	−0.0114 (18)
C23	0.046 (2)	0.049 (3)	0.039 (2)	−0.011 (2)	0.0039 (19)	−0.0012 (19)
C24	0.047 (2)	0.051 (2)	0.039 (2)	−0.0163 (19)	0.0024 (18)	−0.0197 (19)
C25	0.044 (2)	0.051 (3)	0.040 (2)	−0.0127 (19)	−0.0010 (18)	−0.0148 (19)
C26	0.039 (2)	0.0300 (18)	0.027 (2)	0.0043 (15)	−0.0162 (15)	−0.0108 (15)
C27	0.042 (2)	0.044 (2)	0.047 (2)	−0.0100 (18)	−0.0098 (18)	−0.0160 (19)
C28	0.051 (2)	0.042 (2)	0.037 (2)	−0.0029 (18)	−0.0148 (18)	−0.0195 (18)
C29	0.039 (2)	0.033 (2)	0.040 (2)	−0.0131 (16)	0.0058 (17)	−0.0140 (17)
C30	0.0283 (18)	0.046 (2)	0.039 (2)	−0.0166 (16)	0.0022 (16)	−0.0122 (17)
C31	0.033 (2)	0.062 (3)	0.044 (2)	−0.0135 (19)	0.0085 (18)	−0.016 (2)
C32	0.0301 (19)	0.051 (2)	0.039 (2)	−0.0189 (17)	0.0034 (16)	−0.0177 (19)
C33	0.040 (2)	0.038 (2)	0.046 (2)	−0.0121 (17)	−0.0106 (18)	−0.0120 (18)
C34	0.0356 (19)	0.0277 (18)	0.0278 (19)	0.0134 (15)	−0.0113 (15)	−0.0027 (15)
C35	0.049 (2)	0.039 (2)	0.049 (2)	0.0045 (18)	−0.016 (2)	−0.0189 (19)
C36	0.071 (3)	0.043 (2)	0.047 (3)	−0.013 (2)	0.006 (2)	−0.021 (2)
C37	0.055 (3)	0.057 (3)	0.056 (3)	−0.016 (2)	0.010 (2)	−0.015 (2)
C38	0.059 (3)	0.042 (2)	0.046 (3)	−0.019 (2)	0.010 (2)	−0.014 (2)
C39	0.049 (3)	0.042 (2)	0.056 (3)	−0.0120 (19)	0.010 (2)	−0.013 (2)
C40	0.050 (3)	0.066 (3)	0.052 (3)	−0.016 (2)	0.013 (2)	−0.014 (2)
Mn1	0.0301 (3)	0.0251 (3)	0.0240 (3)	−0.0052 (2)	−0.0064 (2)	−0.0018 (2)
Mn2	0.0299 (4)	0.0246 (4)	0.0239 (4)	−0.0050 (3)	−0.0071 (3)	−0.0112 (3)
Mn3	0.0292 (4)	0.0309 (4)	0.0261 (4)	−0.0026 (3)	−0.0083 (3)	−0.0035 (3)
Mn4	0.0406 (3)	0.0370 (3)	0.0321 (3)	−0.0148 (3)	0.0093 (2)	−0.0109 (2)
N1	0.0445 (18)	0.0275 (16)	0.0385 (18)	−0.0123 (14)	0.0072 (14)	−0.0129 (14)
N2	0.0469 (19)	0.0444 (19)	0.0372 (18)	−0.0177 (16)	−0.0089 (15)	−0.0159 (15)
N3	0.047 (2)	0.0424 (19)	0.0396 (19)	0.0116 (15)	−0.0065 (15)	−0.0165 (15)
N4	0.054 (2)	0.044 (2)	0.048 (2)	−0.0185 (17)	0.0029 (17)	−0.0113 (17)
O1	0.0381 (14)	0.0396 (15)	0.0329 (14)	−0.0066 (12)	0.0004 (11)	−0.0112 (12)
O2	0.0400 (15)	0.0368 (15)	0.0369 (15)	−0.0115 (12)	−0.0087 (11)	−0.0082 (12)
O3	0.0372 (14)	0.0347 (14)	0.0344 (14)	−0.0099 (11)	−0.0087 (11)	−0.0058 (11)
O4	0.0360 (13)	0.0357 (14)	0.0301 (13)	−0.0021 (11)	−0.0055 (11)	−0.0128 (11)
O5	0.0381 (14)	0.0350 (14)	0.0274 (13)	−0.0129 (11)	−0.0063 (11)	−0.0040 (11)
O6	0.0331 (13)	0.0287 (13)	0.0240 (12)	−0.0068 (10)	−0.0074 (10)	−0.0023 (10)
O7	0.0408 (15)	0.0301 (14)	0.0405 (15)	−0.0013 (11)	0.0008 (12)	−0.0097 (11)
O8	0.0446 (15)	0.0409 (15)	0.0323 (14)	−0.0141 (12)	0.0087 (12)	−0.0135 (12)
O9	0.0293 (13)	0.0338 (13)	0.0347 (14)	0.0074 (10)	−0.0119 (11)	−0.0122 (11)
O10	0.0375 (14)	0.0408 (15)	0.0361 (15)	−0.0092 (12)	−0.0079 (11)	−0.0162 (12)
O11	0.0360 (14)	0.0363 (15)	0.0410 (15)	−0.0010 (11)	−0.0112 (12)	−0.0044 (12)
O12	0.0509 (17)	0.0417 (16)	0.0315 (16)	0.0029 (13)	−0.0121 (12)	−0.0112 (12)
O13	0.0393 (14)	0.0394 (14)	0.0297 (13)	−0.0125 (12)	0.0011 (11)	−0.0108 (11)
O14	0.0344 (14)	0.0505 (17)	0.0455 (17)	−0.0112 (12)	0.0062 (12)	−0.0178 (14)
O15	0.0409 (15)	0.0409 (15)	0.0379 (15)	−0.0148 (12)	−0.0060 (12)	−0.0093 (12)
O16	0.0531 (17)	0.0441 (16)	0.0507 (18)	−0.0239 (14)	0.0180 (14)	−0.0141 (14)
O17	0.058 (4)	0.046 (4)	0.051 (4)	−0.019 (3)	0.003 (3)	−0.010 (3)

### Geometric parameters (Å, °)

**Table:**

C1—O1	1.262 (5)	C28—H28A	0.9600
C1—O2	1.267 (5)	C29—O13	1.268 (5)
C1—C2	1.465 (5)	C29—O14	1.315 (5)
C2—C3	1.371 (5)	C29—C30	1.485 (6)
C2—C7	1.439 (5)	C30—C31	1.336 (6)
C3—C4	1.410 (5)	C30—C32^ii^	1.412 (5)
C3—H3	0.9300	C31—C32	1.370 (6)
C4—C5	1.390 (5)	C31—H31	0.9300
C4—H4	0.9300	C32—C30^ii^	1.412 (5)
C5—C6	1.415 (5)	C32—H32	0.9300
C5—C8	1.468 (5)	C33—C34	1.543 (5)
C6—C7	1.387 (5)	C33—H33A	0.9600
C6—H6	0.9300	C33—H33B	0.9600
C7—H7	0.9300	C33—H33C	0.9600
C8—O4	1.258 (4)	C34—O15	1.240 (4)
C8—O3	1.289 (5)	C34—N3	1.413 (5)
C9—O6	1.261 (5)	C35—N3	1.513 (5)
C9—O5	1.305 (5)	C35—H35A	0.9600
C9—C10	1.515 (5)	C35—H35B	0.9600
C10—C11	1.373 (5)	C35—H35C	0.9600
C10—C15	1.408 (5)	C36—N3	1.501 (6)
C11—C12	1.389 (5)	C36—H36B	0.9600
C11—H11	0.9300	C36—H36C	0.9600
C12—C13	1.372 (6)	C36—H36A	0.9600
C12—H12	0.9300	C37—C38	1.555 (7)
C13—C14	1.408 (6)	C37—H37B	0.9600
C13—C16	1.525 (5)	C37—H37C	0.9600
C14—C15	1.379 (5)	C37—H37A	0.9600
C14—H14	0.9300	C38—O16	1.265 (5)
C15—H15	0.9300	C38—N4	1.435 (6)
C16—O8	1.251 (5)	C39—N4	1.529 (6)
C16—O7	1.263 (5)	C39—H39A	0.9600
C17—O10	1.251 (4)	C39—H39B	0.9600
C17—O9	1.309 (4)	C39—H39C	0.9600
C17—C18	1.491 (5)	C40—N4	1.506 (5)
C18—C20^i^	1.363 (5)	C40—H40A	0.9600
C18—C19	1.387 (5)	C40—H40B	0.9600
C19—C20	1.405 (5)	C40—H40C	0.9600
C19—H19	0.9300	Mn1—O6^iii^	2.100 (3)
C20—C18^i^	1.363 (5)	Mn1—O1	2.141 (3)
C20—H20	0.9300	Mn1—O12	2.193 (3)
C21—C22	1.462 (5)	Mn1—O9	2.221 (3)
C21—H21A	0.9600	Mn1—O11	2.233 (3)
C21—H21B	0.9600	Mn1—O10	2.389 (3)
C21—H21C	0.9600	Mn2—O2^iii^	2.145 (3)
C22—O11	1.291 (5)	Mn2—O2	2.145 (3)
C22—N1	1.354 (5)	Mn2—O5	2.149 (3)
C23—N1	1.435 (5)	Mn2—O5^iii^	2.149 (3)
C23—H23A	0.9600	Mn2—O9^iii^	2.208 (3)
C23—H23B	0.9600	Mn2—O9	2.208 (3)
C23—H23C	0.9600	Mn3—O7^iv^	2.112 (3)
C24—N1	1.461 (5)	Mn3—O7^v^	2.112 (3)
C24—H24A	0.9600	Mn3—O4^vi^	2.134 (3)
C24—H24B	0.9600	Mn3—O4	2.134 (3)
C24—H24C	0.9600	Mn3—O13	2.192 (3)
C25—C26	1.515 (5)	Mn3—O13^vi^	2.192 (3)
C25—H25A	0.9600	Mn4—O8^v^	2.095 (3)
C25—H25B	0.9600	Mn4—O15	2.162 (3)
C25—H25C	0.9600	Mn4—O3	2.163 (3)
C26—O12	1.087 (4)	Mn4—O16	2.192 (3)
C26—N2	1.441 (5)	Mn4—O14	2.283 (3)
C27—N2	1.484 (5)	Mn4—O13	2.344 (3)
C27—H27A	0.9600	O6—Mn1^iii^	2.100 (3)
C27—H27B	0.9600	O7—Mn3^vii^	2.112 (3)
C27—H27C	0.9600	O8—Mn4^v^	2.095 (3)
C28—N2	1.499 (5)	O17—O17^viii^	1.179 (11)
C28—H28B	0.9600	O17—H17C	0.8500
C28—H28C	0.9600	O17—H17B	0.8500
			
O1—C1—O2	123.2 (3)	N3—C35—H35C	109.5
O1—C1—C2	117.5 (3)	H35A—C35—H35C	109.5
O2—C1—C2	119.3 (3)	H35B—C35—H35C	109.5
C3—C2—C7	117.1 (3)	N3—C36—H36B	109.5
C3—C2—C1	122.8 (4)	N3—C36—H36C	109.5
C7—C2—C1	120.1 (3)	H36B—C36—H36C	109.5
C2—C3—C4	121.3 (4)	N3—C36—H36A	109.5
C2—C3—H3	119.3	H36B—C36—H36A	109.5
C4—C3—H3	119.3	H36C—C36—H36A	109.5
C5—C4—C3	122.2 (4)	C38—C37—H37B	109.5
C5—C4—H4	118.9	C38—C37—H37C	109.5
C3—C4—H4	118.9	H37B—C37—H37C	109.5
C4—C5—C6	117.0 (3)	C38—C37—H37A	109.5
C4—C5—C8	124.7 (3)	H37B—C37—H37A	109.5
C6—C5—C8	118.3 (3)	H37C—C37—H37A	109.5
C7—C6—C5	120.9 (3)	O16—C38—N4	121.2 (4)
C7—C6—H6	119.5	O16—C38—C37	112.0 (4)
C5—C6—H6	119.5	N4—C38—C37	126.5 (4)
C6—C7—C2	121.2 (3)	N4—C39—H39A	109.5
C6—C7—H7	119.4	N4—C39—H39B	109.5
C2—C7—H7	119.4	H39A—C39—H39B	109.5
O4—C8—O3	124.2 (3)	N4—C39—H39C	109.5
O4—C8—C5	120.7 (3)	H39A—C39—H39C	109.5
O3—C8—C5	114.9 (3)	H39B—C39—H39C	109.5
O6—C9—O5	123.2 (3)	N4—C40—H40A	109.5
O6—C9—C10	122.5 (3)	N4—C40—H40B	109.5
O5—C9—C10	114.3 (3)	H40A—C40—H40B	109.5
C11—C10—C15	120.8 (3)	N4—C40—H40C	109.5
C11—C10—C9	122.6 (4)	H40A—C40—H40C	109.5
C15—C10—C9	116.7 (3)	H40B—C40—H40C	109.5
C10—C11—C12	120.4 (3)	O6^iii^—Mn1—O1	103.76 (12)
C10—C11—H11	119.8	O6^iii^—Mn1—O12	86.16 (12)
C12—C11—H11	119.8	O1—Mn1—O12	97.03 (12)
C13—C12—C11	120.8 (4)	O6^iii^—Mn1—O9	100.39 (11)
C13—C12—H12	119.6	O1—Mn1—O9	96.44 (12)
C11—C12—H12	119.6	O12—Mn1—O9	163.16 (10)
C12—C13—C14	117.8 (3)	O6^iii^—Mn1—O11	167.36 (10)
C12—C13—C16	119.1 (4)	O1—Mn1—O11	87.73 (12)
C14—C13—C16	123.1 (4)	O12—Mn1—O11	87.22 (12)
C15—C14—C13	122.7 (4)	O9—Mn1—O11	83.26 (11)
C15—C14—H14	118.7	O6^iii^—Mn1—O10	92.02 (11)
C13—C14—H14	118.7	O1—Mn1—O10	151.75 (10)
C14—C15—C10	117.3 (3)	O12—Mn1—O10	107.42 (11)
C14—C15—H15	121.3	O9—Mn1—O10	57.22 (10)
C10—C15—H15	121.3	O11—Mn1—O10	79.71 (11)
O8—C16—O7	126.6 (3)	O2^iii^—Mn2—O2	180.000 (1)
O8—C16—C13	116.5 (4)	O2^iii^—Mn2—O5	93.06 (10)
O7—C16—C13	116.6 (3)	O2—Mn2—O5	86.94 (10)
O10—C17—O9	119.6 (3)	O2^iii^—Mn2—O5^iii^	86.94 (10)
O10—C17—C18	122.2 (3)	O2—Mn2—O5^iii^	93.06 (10)
O9—C17—C18	118.1 (3)	O5—Mn2—O5^iii^	180.000 (1)
C20^i^—C18—C19	119.7 (3)	O2^iii^—Mn2—O9^iii^	88.32 (12)
C20^i^—C18—C17	121.2 (3)	O2—Mn2—O9^iii^	91.68 (12)
C19—C18—C17	119.0 (3)	O5—Mn2—O9^iii^	89.33 (10)
C18—C19—C20	119.9 (4)	O5^iii^—Mn2—O9^iii^	90.67 (10)
C18—C19—H19	120.1	O2^iii^—Mn2—O9	91.68 (12)
C20—C19—H19	120.1	O2—Mn2—O9	88.32 (12)
C18^i^—C20—C19	120.4 (3)	O5—Mn2—O9	90.67 (10)
C18^i^—C20—H20	119.8	O5^iii^—Mn2—O9	89.33 (10)
C19—C20—H20	119.8	O9^iii^—Mn2—O9	180.0
C22—C21—H21A	109.5	O7^iv^—Mn3—O7^v^	180.000 (1)
C22—C21—H21B	109.5	O7^iv^—Mn3—O4^vi^	91.32 (10)
H21A—C21—H21B	109.5	O7^v^—Mn3—O4^vi^	88.68 (10)
C22—C21—H21C	109.5	O7^iv^—Mn3—O4	88.68 (10)
H21A—C21—H21C	109.5	O7^v^—Mn3—O4	91.32 (10)
H21B—C21—H21C	109.5	O4^vi^—Mn3—O4	180.0
O11—C22—N1	117.5 (3)	O7^iv^—Mn3—O13	92.39 (12)
O11—C22—C21	122.2 (4)	O7^v^—Mn3—O13	87.61 (12)
N1—C22—C21	120.3 (4)	O4^vi^—Mn3—O13	89.45 (10)
N1—C23—H23A	109.5	O4—Mn3—O13	90.55 (10)
N1—C23—H23B	109.5	O7^iv^—Mn3—O13^vi^	87.61 (12)
H23A—C23—H23B	109.5	O7^v^—Mn3—O13^vi^	92.39 (12)
N1—C23—H23C	109.5	O4^vi^—Mn3—O13^vi^	90.55 (10)
H23A—C23—H23C	109.5	O4—Mn3—O13^vi^	89.45 (10)
H23B—C23—H23C	109.5	O13—Mn3—O13^vi^	180.000 (1)
N1—C24—H24A	109.5	O8^v^—Mn4—O15	112.11 (12)
N1—C24—H24B	109.5	O8^v^—Mn4—O3	95.84 (11)
H24A—C24—H24B	109.5	O15—Mn4—O3	91.37 (12)
N1—C24—H24C	109.5	O8^v^—Mn4—O16	86.47 (12)
H24A—C24—H24C	109.5	O15—Mn4—O16	83.87 (12)
H24B—C24—H24C	109.5	O3—Mn4—O16	175.21 (11)
C26—C25—H25A	109.5	O8^v^—Mn4—O14	149.65 (11)
C26—C25—H25B	109.5	O15—Mn4—O14	95.14 (11)
H25A—C25—H25B	109.5	O3—Mn4—O14	96.63 (11)
C26—C25—H25C	109.5	O16—Mn4—O14	83.32 (12)
H25A—C25—H25C	109.5	O8^v^—Mn4—O13	93.91 (12)
H25B—C25—H25C	109.5	O15—Mn4—O13	151.48 (10)
O12—C26—N2	115.4 (4)	O3—Mn4—O13	97.61 (12)
O12—C26—C25	123.0 (4)	O16—Mn4—O13	86.39 (12)
N2—C26—C25	121.3 (3)	O14—Mn4—O13	57.05 (10)
N2—C27—H27A	109.5	C22—N1—C23	124.7 (3)
N2—C27—H27B	109.5	C22—N1—C24	118.2 (3)
H27A—C27—H27B	109.5	C23—N1—C24	116.8 (3)
N2—C27—H27C	109.5	C26—N2—C27	119.0 (3)
H27A—C27—H27C	109.5	C26—N2—C28	118.4 (3)
H27B—C27—H27C	109.5	C27—N2—C28	121.8 (3)
N2—C28—H28B	109.5	C34—N3—C36	119.8 (3)
N2—C28—H28C	109.5	C34—N3—C35	123.4 (3)
H28B—C28—H28C	109.5	C36—N3—C35	116.6 (3)
N2—C28—H28A	109.5	C38—N4—C40	117.4 (4)
H28B—C28—H28A	109.5	C38—N4—C39	122.2 (3)
H28C—C28—H28A	109.5	C40—N4—C39	120.4 (4)
O13—C29—O14	117.6 (3)	C1—O1—Mn1	124.7 (2)
O13—C29—C30	121.1 (4)	C1—O2—Mn2	143.9 (2)
O14—C29—C30	121.1 (4)	C8—O3—Mn4	132.4 (2)
C31—C30—C32^ii^	121.0 (4)	C8—O4—Mn3	136.6 (2)
C31—C30—C29	122.6 (4)	C9—O5—Mn2	141.5 (2)
C32^ii^—C30—C29	116.2 (4)	C9—O6—Mn1^iii^	130.9 (2)
C30—C31—C32	120.1 (4)	C16—O7—Mn3^vii^	142.6 (3)
C30—C31—H31	120.0	C16—O8—Mn4^v^	124.3 (3)
C32—C31—H31	120.0	C17—O9—Mn2	133.0 (2)
C31—C32—C30^ii^	118.7 (4)	C17—O9—Mn1	94.1 (2)
C31—C32—H32	120.6	Mn2—O9—Mn1	108.62 (11)
C30^ii^—C32—H32	120.6	C17—O10—Mn1	88.0 (2)
C34—C33—H33A	109.7	C22—O11—Mn1	125.1 (2)
C34—C33—H33B	108.9	C26—O12—Mn1	130.9 (3)
H33A—C33—H33B	109.5	C29—O13—Mn3	131.9 (2)
C34—C33—H33C	109.8	C29—O13—Mn4	91.6 (2)
H33A—C33—H33C	109.5	Mn3—O13—Mn4	109.57 (12)
H33B—C33—H33C	109.5	C29—O14—Mn4	93.1 (2)
O15—C34—N3	121.7 (3)	C34—O15—Mn4	144.9 (3)
O15—C34—C33	121.3 (3)	C38—O16—Mn4	152.1 (3)
N3—C34—C33	116.9 (3)	O17^viii^—O17—H17C	142.0
N3—C35—H35A	109.5	O17^viii^—O17—H17B	72.4
N3—C35—H35B	109.5	H17C—O17—H17B	111.4
H35A—C35—H35B	109.5		
			
O1—C1—C2—C3	4.4 (6)	O2—Mn2—O5—C9	132.3 (4)
O2—C1—C2—C3	−174.9 (3)	O9^iii^—Mn2—O5—C9	40.6 (4)
O1—C1—C2—C7	−174.6 (3)	O9—Mn2—O5—C9	−139.4 (4)
O2—C1—C2—C7	6.1 (6)	O5—C9—O6—Mn1^iii^	−9.2 (6)
C7—C2—C3—C4	0.3 (5)	C10—C9—O6—Mn1^iii^	172.9 (3)
C1—C2—C3—C4	−178.7 (4)	O8—C16—O7—Mn3^vii^	45.6 (7)
C2—C3—C4—C5	0.8 (6)	C13—C16—O7—Mn3^vii^	−128.3 (4)
C3—C4—C5—C6	1.8 (6)	O7—C16—O8—Mn4^v^	−14.4 (6)
C3—C4—C5—C8	179.4 (4)	C13—C16—O8—Mn4^v^	159.5 (3)
C4—C5—C6—C7	−5.7 (6)	O10—C17—O9—Mn2	109.5 (4)
C8—C5—C6—C7	176.6 (4)	C18—C17—O9—Mn2	−73.8 (4)
C5—C6—C7—C2	7.0 (6)	O10—C17—O9—Mn1	−10.8 (3)
C3—C2—C7—C6	−4.2 (6)	C18—C17—O9—Mn1	165.9 (3)
C1—C2—C7—C6	174.8 (4)	O2^iii^—Mn2—O9—C17	21.5 (3)
C4—C5—C8—O4	−171.6 (4)	O2—Mn2—O9—C17	−158.5 (3)
C6—C5—C8—O4	6.0 (6)	O5—Mn2—O9—C17	114.6 (3)
C4—C5—C8—O3	12.4 (6)	O5^iii^—Mn2—O9—C17	−65.4 (3)
C6—C5—C8—O3	−170.1 (4)	O2^iii^—Mn2—O9—Mn1	136.19 (12)
O6—C9—C10—C11	−172.3 (4)	O2—Mn2—O9—Mn1	−43.81 (12)
O5—C9—C10—C11	9.6 (6)	O5—Mn2—O9—Mn1	−130.74 (11)
O6—C9—C10—C15	7.4 (6)	O5^iii^—Mn2—O9—Mn1	49.26 (11)
O5—C9—C10—C15	−170.6 (3)	O6^iii^—Mn1—O9—C17	91.53 (19)
C15—C10—C11—C12	0.3 (6)	O1—Mn1—O9—C17	−163.16 (19)
C9—C10—C11—C12	−180.0 (4)	O12—Mn1—O9—C17	−20.2 (4)
C10—C11—C12—C13	4.9 (6)	O11—Mn1—O9—C17	−76.23 (19)
C11—C12—C13—C14	−6.9 (6)	O10—Mn1—O9—C17	5.85 (17)
C11—C12—C13—C16	173.4 (4)	O6^iii^—Mn1—O9—Mn2	−46.66 (12)
C12—C13—C14—C15	4.0 (6)	O1—Mn1—O9—Mn2	58.65 (13)
C16—C13—C14—C15	−176.3 (4)	O12—Mn1—O9—Mn2	−158.4 (3)
C13—C14—C15—C10	0.9 (6)	O11—Mn1—O9—Mn2	145.59 (12)
C11—C10—C15—C14	−3.1 (6)	O10—Mn1—O9—Mn2	−132.33 (15)
C9—C10—C15—C14	177.2 (3)	O9—C17—O10—Mn1	10.1 (3)
C12—C13—C16—O8	−167.3 (4)	C18—C17—O10—Mn1	−166.5 (3)
C14—C13—C16—O8	13.0 (6)	O6^iii^—Mn1—O10—C17	−107.2 (2)
C12—C13—C16—O7	7.3 (6)	O1—Mn1—O10—C17	17.5 (3)
C14—C13—C16—O7	−172.4 (4)	O12—Mn1—O10—C17	166.22 (19)
O10—C17—C18—C20^i^	169.4 (3)	O9—Mn1—O10—C17	−6.11 (18)
O9—C17—C18—C20^i^	−7.3 (5)	O11—Mn1—O10—C17	82.5 (2)
O10—C17—C18—C19	−8.3 (5)	N1—C22—O11—Mn1	113.1 (3)
O9—C17—C18—C19	175.0 (3)	C21—C22—O11—Mn1	−68.1 (5)
C20^i^—C18—C19—C20	−2.1 (6)	O6^iii^—Mn1—O11—C22	151.8 (4)
C17—C18—C19—C20	175.7 (3)	O1—Mn1—O11—C22	−3.8 (3)
C18—C19—C20—C18^i^	2.1 (6)	O12—Mn1—O11—C22	93.4 (3)
O13—C29—C30—C31	14.3 (6)	O9—Mn1—O11—C22	−100.6 (3)
O14—C29—C30—C31	−161.4 (4)	O10—Mn1—O11—C22	−158.4 (3)
O13—C29—C30—C32^ii^	−170.6 (3)	N2—C26—O12—Mn1	153.3 (3)
O14—C29—C30—C32^ii^	13.7 (5)	C25—C26—O12—Mn1	−32.7 (6)
C32^ii^—C30—C31—C32	5.4 (7)	O6^iii^—Mn1—O12—C26	−39.6 (4)
C29—C30—C31—C32	−179.7 (4)	O1—Mn1—O12—C26	−143.1 (4)
C30—C31—C32—C30^ii^	−5.2 (7)	O9—Mn1—O12—C26	74.1 (5)
O11—C22—N1—C23	−175.1 (4)	O11—Mn1—O12—C26	129.6 (4)
C21—C22—N1—C23	6.0 (6)	O10—Mn1—O12—C26	51.3 (4)
O11—C22—N1—C24	−2.1 (5)	O14—C29—O13—Mn3	−110.7 (3)
C21—C22—N1—C24	179.0 (4)	C30—C29—O13—Mn3	73.4 (4)
O12—C26—N2—C27	−18.1 (5)	O14—C29—O13—Mn4	7.7 (3)
C25—C26—N2—C27	167.8 (4)	C30—C29—O13—Mn4	−168.2 (3)
O12—C26—N2—C28	152.3 (4)	O7^iv^—Mn3—O13—C29	−26.8 (3)
C25—C26—N2—C28	−21.8 (5)	O7^v^—Mn3—O13—C29	153.2 (3)
O15—C34—N3—C36	0.1 (5)	O4^vi^—Mn3—O13—C29	−118.1 (3)
C33—C34—N3—C36	177.7 (3)	O4—Mn3—O13—C29	61.9 (3)
O15—C34—N3—C35	174.9 (3)	O7^iv^—Mn3—O13—Mn4	−137.83 (12)
C33—C34—N3—C35	−7.5 (5)	O7^v^—Mn3—O13—Mn4	42.17 (12)
O16—C38—N4—C40	−172.7 (4)	O4^vi^—Mn3—O13—Mn4	130.88 (12)
C37—C38—N4—C40	0.9 (6)	O4—Mn3—O13—Mn4	−49.12 (12)
O16—C38—N4—C39	6.6 (6)	O8^v^—Mn4—O13—C29	165.9 (2)
C37—C38—N4—C39	−179.8 (4)	O15—Mn4—O13—C29	9.6 (3)
O2—C1—O1—Mn1	−12.4 (5)	O3—Mn4—O13—C29	−97.7 (2)
C2—C1—O1—Mn1	168.4 (2)	O16—Mn4—O13—C29	79.7 (2)
O6^iii^—Mn1—O1—C1	69.4 (3)	O14—Mn4—O13—C29	−4.7 (2)
O12—Mn1—O1—C1	157.2 (3)	O8^v^—Mn4—O13—Mn3	−58.07 (13)
O9—Mn1—O1—C1	−33.0 (3)	O15—Mn4—O13—Mn3	145.60 (17)
O11—Mn1—O1—C1	−115.9 (3)	O3—Mn4—O13—Mn3	38.36 (13)
O10—Mn1—O1—C1	−52.7 (4)	O16—Mn4—O13—Mn3	−144.27 (13)
O1—C1—O2—Mn2	40.3 (6)	O14—Mn4—O13—Mn3	131.35 (15)
C2—C1—O2—Mn2	−140.4 (3)	O13—C29—O14—Mn4	−7.9 (3)
O5—Mn2—O2—C1	86.5 (4)	C30—C29—O14—Mn4	168.0 (3)
O5^iii^—Mn2—O2—C1	−93.5 (4)	O8^v^—Mn4—O14—C29	−14.4 (3)
O9^iii^—Mn2—O2—C1	175.8 (4)	O15—Mn4—O14—C29	−168.7 (2)
O9—Mn2—O2—C1	−4.2 (4)	O3—Mn4—O14—C29	99.3 (2)
O4—C8—O3—Mn4	3.0 (6)	O16—Mn4—O14—C29	−85.5 (2)
C5—C8—O3—Mn4	178.9 (2)	O13—Mn4—O14—C29	4.50 (19)
O8^v^—Mn4—O3—C8	84.9 (3)	N3—C34—O15—Mn4	144.5 (3)
O15—Mn4—O3—C8	−162.7 (3)	C33—C34—O15—Mn4	−33.1 (6)
O14—Mn4—O3—C8	−67.4 (3)	O8^v^—Mn4—O15—C34	124.5 (4)
O13—Mn4—O3—C8	−9.9 (3)	O3—Mn4—O15—C34	27.6 (4)
O3—C8—O4—Mn3	−36.0 (6)	O16—Mn4—O15—C34	−151.8 (4)
C5—C8—O4—Mn3	148.4 (3)	O14—Mn4—O15—C34	−69.1 (4)
O7^iv^—Mn3—O4—C8	153.1 (4)	O13—Mn4—O15—C34	−81.1 (5)
O7^v^—Mn3—O4—C8	−26.9 (4)	N4—C38—O16—Mn4	60.4 (9)
O13—Mn3—O4—C8	60.7 (4)	C37—C38—O16—Mn4	−114.0 (6)
O13^vi^—Mn3—O4—C8	−119.3 (4)	O8^v^—Mn4—O16—C38	−85.6 (7)
O6—C9—O5—Mn2	−8.0 (7)	O15—Mn4—O16—C38	161.7 (7)
C10—C9—O5—Mn2	170.1 (3)	O14—Mn4—O16—C38	65.8 (7)
O2^iii^—Mn2—O5—C9	−47.7 (4)	O13—Mn4—O16—C38	8.5 (7)

Symmetry codes: (i) −*x*, −*y*+1, −*z*+1; (ii) −*x*+3, −*y*+2, −*z*; (iii) −*x*+1, −*y*+1, −*z*+1; (iv) *x*+1, *y*, *z*; (v) −*x*+1, −*y*+2, −*z*; (vi) −*x*+2, −*y*+2, −*z*; (vii) *x*−1, *y*, *z*; (viii) −*x*+2, −*y*+3, −*z*.

### Hydrogen-bond geometry (Å, °)

**Table:**

*D*—H···*A*	*D*—H	H···*A*	*D*···*A*	*D*—H···*A*
O17—H17B···O16^viii^	0.85	2.61	3.236 (7)	131
O17—H17B···N4^viii^	0.85	2.62	3.463 (8)	170

Symmetry codes: (viii) −*x*+2, −*y*+3, −*z*.
